# The Association Between Lipedema and Attention-Deficit/Hyperactivity Disorder

**DOI:** 10.7759/cureus.35570

**Published:** 2023-02-28

**Authors:** Alexandre C Amato, Juliana L Amato, Daniel A Benitti

**Affiliations:** 1 Department of Vascular Surgery, Amato - Instituto de Medicina Avançada, São Paulo, BRA; 2 Department of Vascular and Endovascular Surgery, Medical Valens Center, São Paulo, BRA

**Keywords:** lymphedema, obesity, adhd, lipedema, questionnaires, prevalence

## Abstract

Introduction

The current study aimed to investigate the overlap between symptoms of lipedema and attention-deficit/hyperactivity disorder (ADHD). Lipedema is a condition that causes abnormal fat accumulation and inflammation in the legs and buttocks, often accompanied by edema and pain. ADHD is a common condition characterized by difficulty paying attention and controlling behavior, affecting the social, academic, and occupational quality of life. The study’s primary objective was to assess the prevalence of ADHD symptoms in a population of women with lipedema symptoms and compare the clinical characteristics.

Method

The study used a lipedema screening questionnaire and the Adult Self-Report Scale (ASRS-18) to assess the prevalence of ADHD in a sample of 354 female volunteers with or without a prior lipedema diagnosis.

Results

Of the lipedema group, 100 (77%) were ASRS positive, and 30 (23%) were ASRS negative. In the group without lipedema, 121 (54%) were ASRS positive, and 103 (46%) were ASRS negative, with a relative risk of 1.424 (p<0.0001).

Conclusion

Our results demonstrate a positive correlation between lipedema and ADHD and suggest that targeted strategies to improve clinic attendance for individuals with ADHD may improve lipedema treatment outcomes. Patients with lipedema symptoms are more likely to have ADHD symptoms.

## Introduction

The current study examined the overlap between lipedema symptoms and attention-deficit/hyperactivity disorder (ADHD). Lipedema is characterized by the abnormal accumulation of fat in the lower limbs, which may be accompanied by complaints of pain and edema when standing up [1,2]. The cause of lipedema is not well understood, but it is known to be linked to inflammation. Lipedema is often mistaken for other conditions, such as obesity, gynoid lipodystrophy, and lymphedema, and is frequently not diagnosed during the first medical visit. Lipedema is more common in women, and imaging tests such as ultrasound [3], magnetic resonance imaging, and computed tomography can confirm the diagnosis. Recently, a self-administered questionnaire was developed with excellent screening accuracy for lipedema [1].

ADHD is a common condition affecting the quality of life for children and adults in social, academic, and occupational contexts. The underlying causes of ADHD are not fully understood. Still, proposed mechanisms include glial activation, neuronal damage and degeneration, increased oxidative stress, reduced neurotrophic support, altered neurotransmitter metabolism, and blood-brain barrier disruption. There is growing evidence that inflammation may also play a role in ADHD. However, this evidence is mainly from observational studies showing a strong co-occurrence of ADHD with inflammatory and autoimmune disorders, studies evaluating serum inflammatory markers, and genetic studies. Inflammation may be an essential factor in the development of ADHD, but further research is needed to confirm this [4]. ADHD is better understood as a multidimensional disorder that often persists into adulthood [5].

This study aims to evaluate the correlation between lipedema and attention-deficit/hyperactivity disorder (ADHD) in the Brazilian female population. The primary objective is to assess the prevalence of ADHD in women with lipedema characteristics. Understanding the prevalence rates of ADHD in this population has direct clinical implications since this subgroup has inflammatory triggers. Additionally, the study will describe the clinical characteristics of those presenting ADHD compared to those not meeting the criteria.

## Materials and methods

We used the 10-question lipedema screening questionnaire previously published [6] and the standardized Adult Self-Report Scale (ASRS-18) questionnaire [7]. Additional data were collected regarding age, weight, and height, and body mass index (BMI) was calculated using self-reported weight and height. They were converted into an online digital version using secure software appropriate for developing and analyzing questionnaires (SurveyMonkey, CA, USA). The questionnaire was disclosed on a non-profit Brazilian lipedema website (www.lipedema.org.br), and it was administered to 354 volunteers with or without a prior lipedema diagnosis. The participants were women over 18 years old who registered on the online survey platform and volunteered. The sampling method used is convenience sampling. Participants who did not digitally sign the consent form were excluded. Questionnaires completed in less than 300 seconds were excluded. A mean time was previously calculated as three minutes [6] for the lipedema questionnaire and less than two minutes [8] for the ASRS.

Screening probability of lipedema

The measure was based on our group’s previously proposed lipedema screening questionnaire [6] and cutoff validation [1]. The lipedema screening questionnaire total score cutoff method was used, with an area under the receiver operating curve (ROC curve) of 0.8615, which can be considered an excellent level of accuracy [1]. We took a conservative approach, aiming to achieve specificity closer to 0.9, setting the cutoff at 12. At that point, the probability of a lipedema diagnosis is 77.8% (95% confidence interval (CI): 64.2%-87.3%), with a sensitivity of 0.46 and specificity of 0.88, as previously proposed [1]. The methodology is based on the sum of points from a self-administered questionnaire in a population survey, indicating the probability of a lipedema diagnosis.

Screening probability of ADHD

All eligible subjects completed the Adult Self-Report Scale (ASRS-18) in Portuguese [7], an 18-item questionnaire on current ADHD symptoms. Volunteers completed the ASRS online just after completing the lipedema screening. Since there is no psychometric data on the ASRS in Brazil, we used the 18-item version instead of the six-item screening version. Shaded boxes in the ASRS were ignored, and the checklist was interpreted in a binary fashion: symptoms reported as often or very often were considered positive. All other frequencies were considered “negative,” as previously suggested by others [5]. Volunteers reporting at least five positive symptoms in the inattention or hyperactivity-impulsivity domain were considered “ASRS positive.” This strategy rendered sensitivity and specificity rates of 0.97 and 0.40, respectively [5].

Statistical analysis

A sample size of 152 questionnaires was calculated to achieve a 95%CI, considering a 5.5% margin of error. Statistical analysis was conducted after the consistency of the data had been checked manually and automatically with software developed explicitly for this analysis using Xojo 4.1 (Xojo, Austin, TX, USA). Descriptive statistics and frequencies were calculated. Correlations between questionnaire variables were assessed using the chi-square test (z-score), Mann-Whitney, and Student’s t-test. We adopted a p<0.05 level of statistical significance for the correlations. The software used for data analysis is MedCalc® Statistical Software version 20.211 (MedCalc Software Ltd., Ostend, Belgium) (https://www.medcalc.org (2023)) and Wizard 2.0.12 (Evan Miller, MA, USA). This study complies with the National Health Council standards set out in resolution 196/96 regulating research involving human beings. It also adheres to the Helsinki Declaration. The institution’s ethical committee approved the study, and all subjects signed an informed consent form before participation.

## Results

The total sample included 354 eligible volunteers who met the inclusion criteria. The mean (±standard deviation (SD)) age of the whole study population was 36.8 (±9.1 SD) years, while the mean (±SD) age of volunteers with the lipedema diagnostic criterion was 38.2 (±9.6 SD) years, which is equivalent. The mean (±SD) height of the whole study population was 163.4 (±6.3 SD) cm, while the mean height of volunteers with the diagnostic criterion was 163.3 (±6.6 SD) cm, which is equivalent. Within the lipedema group, 100 (77%) were ASRS positive, and 30 (23%) were ASRS negative. Of the subgroup of volunteers without diagnostic criteria for lipedema, 121 (54%) were ASRS positive, and 103 (46%) were ASRS negative (Table 1).

**Table 1:** Demographic and clinical characteristics of the sample: health-related factors associated with lipedema in volunteers with scores over the cutoff compared with those below the cutoff. SD: standard deviation, BMI: body mass index, ASRS: Adult Self-Report Scale, +: positive, -: negative

	Volunteers with diagnostic criteria for lipedema	Volunteers without diagnostic criteria for lipedema	Total	Statistics (chi-square)
Number	130 (36.7%±5)	224 (63.3%±5)	354	Unequal proportions p<0.001
Age (years)	38.2 (±9.6 SD)	36.1 (±8.8 SD)	36.8 (±9.1 SD)	Independent p=0.251
BMI (kg/m^2^)	30.9 (±4.9 SD)	28.5 (±5.4 SD)	29.4 (±5.3 SD)	Independent p<0.001
Weight (kg)	82.7 (±14.7 SD)	76.3 (±15.2 SD)	78.6 (±15.3 SD)	Independent p<0.001
Height (cm)	163.3 (±6.6 SD)	163.4 (±6.1 SD)	163.4 (±6.3 SD)	Independent p=0.317
ASRS +	100 (76.9%±7.15)	121 (54%±6.45)	221 (62.4%±5)	z-score p<0.001
ASRS -	30 (23.1%±7.15)	103 (46%±6.45)	133 (37.6%±5)	z-score p<0.001

There was a 35% dropout rate (less than 300 seconds and fill-out mistakes) during the completion of the questionnaire (n=190, total 547), and the mean time taken to respond was 482 seconds. It was observed that 36.7%±5 (n=130, 95%CI, unequal proportions, z-score p<0.001) of the study population met the lipedema diagnosis criteria, while 62.4%±5 (n=221, 95%CI, unequal proportions, z-score p<0.001) were ASRS positive, and 37.6% (n=133) were ASRS negative, with a relative risk of 1.424 (95%CI: 1.2218-1.6598, p<0.0001) (Table 2).

**Table 2:** Demographic and clinical characteristics of the ASRS groups: health-related factors associated with ADHD in volunteers with scores over the cutoff compared with those below the cutoff. ADHD: attention-deficit/hyperactivity disorder, ASRS: Adult Self-Report Scale, BMI: body mass index, SD: standard deviation

	ASRS positive	ASRS negative	Statistics (Mann-Whitney)
Age (years)	36.2 (±8.8 SD)	37.9 (±9.6 SD)	Equal medians p=0.1794
BMI (kg/m^2^)	29.3 (±4.99 SD)	29.5 (±5.9 SD)	Equal medians p=0.7652
Weight (kg)	78.5 (±14.2 SD)	78.7 (±16.9 SD)	Equal medians p=0.670
Height (cm)	163.5 (±6.0 SD)	163.2 (±6.7 SD)	Equal medians p=0.6961

**Figure 1:**
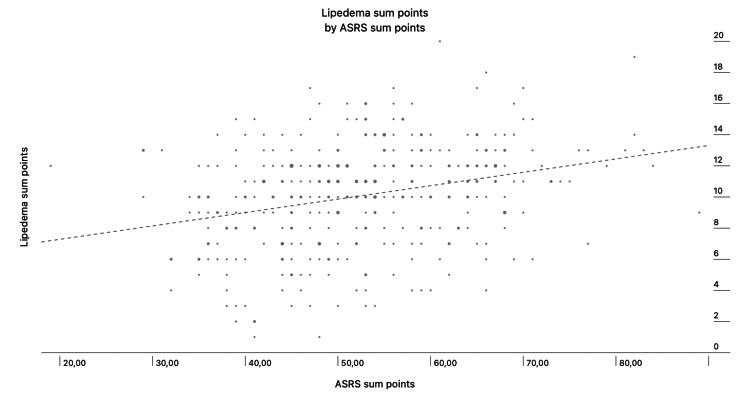
Scatterplot visualizing the relationship between lipedema sum points and ASRS sum points. Pearson correlation, p<0.001, positive correlation ASRS: Adult Self-Report Scale

When evaluating the sum of points of the lipedema and ASRS questionnaire, a positive correlation was found and plotted in Figure 1 (Pearson correlation, p<0.001).

## Discussion

ADHD is a psychiatric disorder that affects approximately 5% of children and has a high genetic component and various potential causes [9]. About 6.76% of adults have ADHD, although there is some variation in the studies on this topic. ADHD symptoms tend to lessen as adults age [10], but this trend was not statistically shown in our data, despite a slightly younger age in the ASRS positive group.

Lipedema is characterized by abnormal fat accumulation in the subcutaneous tissue, leading to inflammation and fibrosis in the affected tissue, affecting 12.3% of women [1,11]. This can cause pain and worsen symptoms. Lipedema seems to have an essential impact on mental health as it has been previously correlated to depressive symptoms and anxiety [1,12-14].

In recent years, there has been increasing research on the potential role of inflammation in developing psychiatric disorders, including ADHD. This is supported by the high co-occurrence of ADHD and inflammatory or autoimmune disorders and biomarkers and genetic associations with ADHD. These findings suggest that various underlying mechanisms, such as altered immune response and shared genetic and environmental factors, may be involved in the relationship between inflammation and ADHD. Higher levels of inflammation during early development may lead to the development of ADHD symptoms. Since ADHD has a strong genetic component, individuals with ADHD may have polymorphisms in genes related to inflammation. While some studies have identified such associations, there is no clear consensus on the specific genes involved. Overall, the evidence suggests that the immune system may play a role in the pathophysiology of ADHD [5]. Given the connection between inflammation and both lipedema and ADHD, as well as the overlap in symptoms between the two conditions, it is worth considering the possibility of concomitance between lipedema and ADHD.

Some patients may also possess cognitive dysfunction concerning measures commonly impaired with ADHD, such as executive function and learning. Early diagnosis and intervention may explain why up to 60% of individuals with ADHD experience partial remission of symptoms and improved cognitive function upon entering adulthood [15]. Conversely, individuals without an ADHD diagnosis but demonstrating decreased cognitive functioning may be less likely to exhibit improvement in response to the same therapeutic interventions or experience similar disease regression.

Many studies on lipedema suggest that surgical treatment is the immediate solution and that removing modified fat tissue can significantly improve patient quality of life. Still, lipedema can be managed clinically [16]. However, some research indicates that lipedema may be connected to other diseases, which may mean that it is a result rather than a cause of these conditions. This highlights the importance of considering a more comprehensive range of factors when evaluating the effectiveness of lipedema surgery.

The similarity between the population groups studied was demonstrated. There was a difference regarding weight and BMI, probably because lipedema can interfere with body weight. Dudek et al. [12]. also used a questionnaire to investigate a group of Polish women with suspected lipedema, estimating mean BMI at 30.8 (±7.1 SD) kg/m^2^, with 76.5% classified as overweight (26.5%) or obese (50%). Another study observed that 67.5% of women with lipedema had BMI greater than 25 kg/m^2^, with a mean BMI of 27 kg/m^2^. Elevated BMI makes diagnosis more difficult because of the complexity of differentiation from common obesity [1]. Although obesity has been implicated with ADHD [17], our study showed no significant difference when comparing ASRS groups, with an equal median (Mann-Whitney, p=0.7652).

Our study demonstrated a positive correlation between lipedema symptoms and ADHD symptoms, meaning that as self-reported lipedema symptoms increase, ASRS symptoms also tend to increase (Figure 1).

ADHD exhibits a diversity of phenotypes, including inattention, impulsivity, or a combination of both. These specific features may not always be accounted for in surgical literature and may potentially influence surgical outcomes [18].

It is also worth noting that individuals with ADHD are at increased risk of comorbid mental health disorders, including mood and anxiety disorders [19]. Anxiety is reported in 61.3% of lipedema patients and depression in 38.7% [1]. This relationship is significant as recent studies have shown that poor cognitive functioning before surgery is closely linked to poor long-term weight loss outcomes following bariatric surgery and may have a similar impact on lipedema treatment [20,21].

Mocanu et al. [18]. found that ADHD patients have lower postoperative follow-up rates than non-ADHD patients following bariatric surgery. Implementing targeted strategies to improve clinical attendance for ADHD patients at risk may improve outcomes and reduce recidivism rates. Additionally, pharmacological treatment of ADHD may improve binge eating and impulsivity through reported improvements in anxiety, time management, and self-awareness, which may positively affect lipedema treatment. Understanding the potential overlap between lipedema and ADHD may aid in developing more effective exercise strategies for managing both conditions [22].

As a standard of care, lipedema surgery should only be considered for patients with controlled mental health conditions who can understand the risks and benefits of surgery and participate in necessary follow-up care.

The current self-report measures for assessing the presence of ADHD and lipedema have limitations. Lipedema inflammation is cyclical [2], so it may influence ADHD more at certain times. The worst time to decide on surgical treatment may be during times of peak inflammation, as inflammation could significantly impact cognitive function at these times. The study was not designed to identify the intensity of inflammation at the testing time.

The lipedema screening questionnaire and ASRS, available in the public domain, can provide a quick and cost-effective means of predicting the likelihood of a diagnosis but cannot give a definitive diagnosis. A previous study found that the ASRS can distinguish between individuals with previously diagnosed ADHD recruited from disability services [23]. Another study used the lipedema screening questionnaire to estimate the prevalence of the disease [1]. However, the literature on the relationship between ADHD and lipedema is not robust, which limits our understanding of more complex interactions between the two conditions. There may also be a discrepancy between self-reported and objective clinical analysis.

Psychiatric disorders are mainly characterized by symptoms and not observable signs, which require interpreting the patient’s report and translating it into diagnostic terms. Self-reflection and self-evaluation can also be problematic and may lead to over- or underreporting of symptoms. Additionally, psychiatric symptoms or clusters of symptoms are often not specific to a particular disorder. Different patient assessment strategies, such as open, structured, or semi-structured interviews, can significantly influence the results, and even small changes in question-wording can affect the responses. While standardized interviews are considered the gold standard for psychiatric diagnosis, efforts to minimize these limitations can involve additional patient-related data, often improving diagnostic accuracy [5].

The sample of volunteers in this study consisted of women who registered on an online survey platform seeking information about lipedema. As a result, this population may not represent the general population, and there may be selection bias. It is possible that individuals with more severe or complex cases of lipedema or ADHD may have been more motivated to participate, which could have influenced the results. Compared to a previous population study that showed that 12.3% of volunteers met the criteria for lipedema diagnosis, our study found a higher rate of 36.7% [1]. Additionally, we did not clinically confirm the Diagnostic and Statistical Manual of Mental Disorders, fourth edition (DSM-IV) criteria for ADHD or clinical lipedema diagnosis, which could also have affected the findings. Mental illnesses often exhibit a range of symptoms and can impact an individual’s functioning in various ways that were not necessarily captured in this study.

These limitations suggest further research on the potential correlation between ADHD and lipedema. That way, we can better understand lipedema’s neurophysiological mechanisms. This includes exploring inflammation’s role in ADHD and lipedema and developing methods for better measuring low-grade chronic inflammation [2]. Incorporating mental health considerations into treatment approaches for lipedema may be beneficial [22], and routine screening for ADHD in lipedema patients may improve clinical treatment strategies. Additionally, developing educational materials on ADHD for medical professionals who treat lipedema could increase awareness of the potential overlap between these conditions and improve diagnosis and treatment.

## Conclusions

In this study, we found a higher prevalence of self-reported symptoms of ADHD in patients with lipedema symptoms. This suggests a potential overlap between the two conditions and highlights the importance of screening for ADHD in patients with lipedema. Implementing targeted strategies to improve clinic attendance for individuals with ADHD may improve lipedema treatment outcomes and reduce variability in results. Furthermore, incorporating mental health considerations, including routine screening for ADHD, into treatment approaches for lipedema may be beneficial. While the limitations of self-reported symptoms should be considered, our study provides insights into the potential correlation between lipedema and ADHD.

## References

[1] Amato AC, Amato FC, Amato JL, Benitti DA (2022). Lipedema prevalence and risk factors in Brazil. J Vasc Bras.

[2] Amato ACM (2020). Is lipedema a unique entity?. EC Clin Med Case Rep.

[3] Amato AC, Saucedo DZ, Santos KD, Benitti DA (2021). Ultrasound criteria for lipedema diagnosis. Phlebology.

[4] Leffa DT, Torres IL, Rohde LA (2018). A review on the role of inflammation in attention-deficit/hyperactivity disorder. Neuroimmunomodulation.

[5] Mattos P, Nazar BP, Tannock R (2018). By the book: ADHD prevalence in medical students varies with analogous methods of addressing DSM items. Braz J Psychiatry.

[6] Amato AC, Amato FC, Benitti DA, Amato LG (2020). Development of a questionnaire and screening model for lipedema. J Vasc Bras.

[7] Mattos P, Segenreich D, Saboya E (2006). Cross-cultural adaptation of the Adult Self-Report Scale to Portuguese for assessing attention-deficit/hyperactivity disorder (ADHD) in adults (Article in Portuguese). Arch Clin Psych (São Paulo).

[8] Kessler RC, Adler L, Ames M (2005). The World Health Organization Adult ADHD Self-Report Scale (ASRS): a short screening scale for use in the general population. Psychol Med.

[9] Choudhry Z, Sengupta SM, Grizenko N, Harvey WJ, Fortier MÈ, Schmitz N, Joober R (2013). Body weight and ADHD: examining the role of self-regulation. PLoS One.

[10] Song P, Zha M, Yang Q, Zhang Y, Li X, Rudan I (2021). The prevalence of adult attention-deficit hyperactivity disorder: a global systematic review and meta-analysis. J Glob Health.

[11] Herbst KL, Kahn LA, Iker E (2021). Standard of care for lipedema in the United States. Phlebology.

[12] Dudek JE, Białaszek W, Gabriel M (2021). Quality of life, its factors, and sociodemographic characteristics of Polish women with lipedema. BMC Womens Health.

[13] Romeijn JR, de Rooij MJ, Janssen L, Martens H (2018). Exploration of patient characteristics and quality of life in patients with lipoedema using a survey. Dermatol Ther (Heidelb).

[14] Torre YS, Wadeea R, Rosas V, Herbst KL (2018). Lipedema: friend and foe. Horm Mol Biol Clin Investig.

[15] Nielsen F, Georgiadou E, Bartsch M, Langenberg S, Müller A, de Zwaan M (2017). Attention deficit hyperactivity disorder prevalence and correlates pre- and post-bariatric surgery: a comparative cross-sectional study. Obes Facts.

[16] Amato AC, Benitti DA (2021). Lipedema can be treated non-surgically: a report of 5 cases. Am J Case Rep.

[17] Cortese S, Vincenzi B (2012). Obesity and ADHD: clinical and neurobiological implications. Curr Top Behav Neurosci.

[18] Mocanu V, Tavakoli I, MacDonald A, Dang JT, Switzer N, Birch DW, Karmali S (2019). The impact of ADHD on outcomes following bariatric surgery: a systematic review and meta-analysis. Obes Surg.

[19] Choi WS, Woo YS, Wang SM, Lim HK, Bahk WM (2022). The prevalence of psychiatric comorbidities in adult ADHD compared with non-ADHD populations: a systematic literature review. PLoS One.

[20] Spitznagel MB, Alosco M, Strain G (2013). Cognitive function predicts 24-month weight loss success after bariatric surgery. Surg Obes Relat Dis.

[21] Spitznagel MB, Alosco M, Galioto R (2014). The role of cognitive function in postoperative weight loss outcomes: 36-month follow-up. Obes Surg.

[22] Amato AC (2022). Exercise method for lipedema: guide for the personal trainer (Book in Portuguese). São Paulo.

[23] Gray S, Woltering S, Mawjee K, Tannock R (2014). The Adult ADHD Self-Report Scale (ASRS): utility in college students with attention-deficit/hyperactivity disorder. PeerJ.

